# Internet use by pregnant women during prenatal care

**DOI:** 10.31744/einstein_journal/2024AO0447

**Published:** 2024-03-21

**Authors:** Carolina Fernandes Giacometti, Giulia Siqueira Galfano, Denis Schapira Wajman, Eduardo Cordioli, Ana Paula Avritscher Beck, Sérgio Podgaec

**Affiliations:** 1 Hospital Israelita Albert Einstein São Paulo SP Brazil Hospital Israelita Albert Einstein, São Paulo, SP, Brazil.

**Keywords:** Prenatal care, Medical informatics, Pregnant women, Internet search, Information-seeking behavior, Consumer health information, Surveys and questionnaires

## Abstract

**Objective:**

The search for medical information on the internet is a part of people's daily lives. Exponential volumes of data are available through various media and platforms. There are several problems related to the ease of creating and accessing medical information on the internet, as evidenced by the quantity of false content and increasing anxiety due to the consumption of these data. In light of this accessibility, it is necessary to understand how people use internet-based medical information and its impact on specific populations. This prospective study aimed to analyze pregnant women's behavior when searching for health-related information on the internet, and how they were influenced by the information.

**Methods:**

Questionnaires were administered to the participants during their immediate puerperium, and their answers were tabulated.

**Results:**

Three hundred and two patients answered the questionnaires. We observed that internet use was frequent, and most patients discussed the findings with their physicians. However, this did not affect the delivery routes.

**Conclusion:**

The search for health information by pregnant women is very prevalent but does not interfere with the delivery route.

## In Brief

Internet impact on prenatal care: Brazilian study found that 92% of pregnant women used the internet to access health information. Fetal development, nutrition, and childbirth were the top search topics. While 88.1% discussed their findings with doctors, the study showed no impact on delivery routes. Caution is required because of potential misinformation. The authors advocate improved guidance and quality control of online health information.

## Highlights

■High internet use: 92% of pregnant women used the internet to access health information during prenatal care.

■Top search topics: fetal development, nutrition, and childbirth were the primary topics of interest.

■Doctor-patient discussions: 88.1% discussed online findings with doctors, emphasizing patient engagement.

■No impact on delivery route: despite extensive internet use, no statistical impact on delivery route choice was observed.

## Graphical abstract

**Figure:**
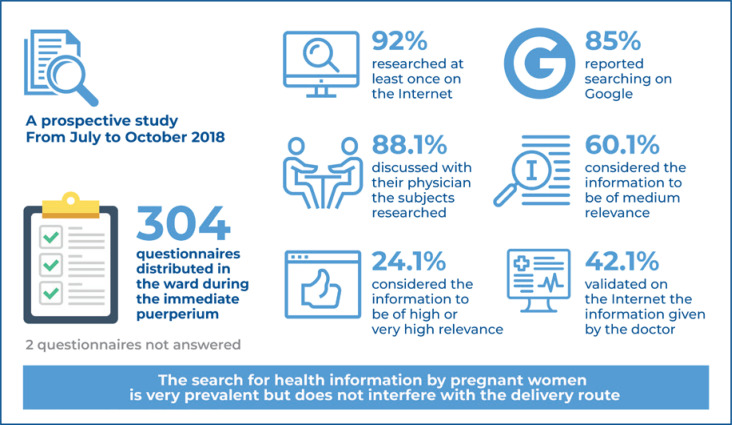

## INTRODUCTION

Commercial use of the internet in Brazil began in 1995, and, as in other countries, the number of internet users has grown exponentially each year.^( 1 )^ A survey by the *Centro Regional para o Desenvolvimento de Estudos sobre a Sociedade da Informação* (CETIC) showed that in 2019 there were 152 million internet users in Brazil, representing 81% of the population over 10 years of age.^( 2 )^ In 2022, there were 5.3 billion users worldwide, representing 66% of the world's population.^( 3 )^ It is estimated that for 80% of American adults, the internet is the main source of health information.^( 4 , 5 )^

The extent of the impact of this topic was evaluated in a study which found that Europeans and Americans had consulted the Internet at least once in the past month regarding a health-related topic.^( 6 )^ Electronic health (e-health) is a trend that aims to improve local, regional, and global health using information and communication technology.^( 7 )^

In Brazil, 26% of the population use a search engine as the first line of medical information when faced with a medical issue, and the number tends to increase with the growth of this communication channel. According to a 2019 Google survey, there was a 17.3% increase in health searches on their platforms.^( 1 , 8 )^

However, as a consequence of easy access and freedom of publication, the volume of health information on the Internet compromises the quality, safety, and reliability of these data. Thus, internet users may be exposed to a large amount of dubious, inconsistent, and inadequate information that can be harmful, promote anxiety, and cause health problems^( 1 , 7 )^ because users lack the technical knowledge to judge the quality and accuracy of the information obtained. According to a British survey, less than one-fifth of the interviewees believed that more than 80% of the information provided on the internet was accurate.^( 9 )^

Studies have shown that internet users seek health-related information primarily at two different times: before contact with health professionals and immediately after their medical consultations.^( 7 , 10 , 11 )^ However, they did not always discuss the knowledge they had acquired through the Internet with their physician.^( 7 )^

For women of reproductive age, the search for medical information is becoming increasingly prevalent, particularly during pregnancy. Research in the United States has shown that three-fourths of all pregnant women search for online information about pregnancy and labor.^( 12 )^ Early studies regarding online research and pregnancy focused on determining whether women trusted the information received.^( 13 )^ Once the studies began focusing on which subjects were searched, it was found that most searches were related to vitamins and nutrition.^( 14 )^ A 2016 systematic review reported that pregnant women accessed health information online at least once a month, and the most searched topics were fetal development and nutrition.^( 15 )^

Another way of using the internet is through forums and support groups, where women exchange experiences and connect with other pregnant women. This has become a common form of interpersonal exchange. Associations such as the American College of Obstetricians and Gynecologists (ACOG) and the Royal College of Obstetricians and Gynecologists (RCOG) note that platforms for e-health and care for pregnant and postpartum women should be supported and implemented to reduce hospital burden and care. From there, interactions arise via the Web between health professionals and pregnant women, which then become another form of relationship. Studies have demonstrated greater paternal engagement during pregnancy when virtual interactions are offered.^( 16 )^

A 2018 review showed the impact of perinatal care through e-health and concluded that most health outcomes for perinatal interventions were generally positive, with the satisfaction of the service provider at around 95%, but regulation was still lacking in many countries, such as the end-to-end encryption of information.^( 17 )^ A 2018 Canadian study reported that owing to geographical diversity (urban and rural), the distribution of health and prenatal care is a challenge; therefore, online platforms that promote health are highly recognized as forms of assistance. It was concluded that the presence of poor-quality and imprecise sites hinder this communication channel, but it is a promising tool.^( 18 )^

Few studies have examined the behavior of women who use the Internet during prenatal care, but it is important to know how they search for issues related to pregnancy and childbirth, which terms are most prevalent, and how they access medical information online. Our goal was to understand how pregnant women in prenatal care use the Internet; this information can be used to improve the approach during consultations, reduce doubts and anxieties related to this period, and provide better care for these individuals.

## OBJECTIVE

To analyze how pregnant women use medical information on the internet and how it impacts that population.

## METHODS

This descriptive cross-sectional study was conducted at the *Hospital Israelita Albert Einstein* in São Paulo between July and October 2018. For data collection, a questionnaire (Appendix 1) was developed to measure Internet use during prenatal care. Women whose deliveries were performed in the maternity hospital received a physical questionnaire in the ward during the immediate puerperium. The interpretation of the questions and completion of the questionnaire were performed by the women without team intervention. Women requiring intensive care and those with unfavorable fetal outcomes were excluded.

The following parameters were evaluated: age, level of education, parity, whether and with what frequency any research on pregnancy/childbirth was performed on the Internet, on which site the search was done, which topics were researched, whether the subject searched for was discussed with a doctor if the information found was validated, and the impact of the information on decision making. The relevance of the information consulted was subjective based on the participant's perception, and no orientation was offered regarding this question.

Regarding the amount of internet access, accessing the internet on 10 or fewer occasions throughout pregnancy was considered "a few times," accessing the internet between 11 and 99 times was "moderate," and accessing the internet on more than 100 occasions was regarded as "often."

Statistical analyses were performed using SAS version 6.11 (Statistical Analysis System Institute Inc., Cary, NC, USA). Initially, a descriptive analysis of the data was performed using the Proc MEANS and Proc FREQ functions of SAS that analyze (i) the mean, including standard deviation, maximum, and minimum, and (ii) frequency, including representativeness in the selected sample, respectively. Relevant characteristics of the population that could help in understanding the profile of the participants in this study ( *e.g.* , parity, age, schooling, and research motive) were also evaluated. In addition, using the information available in table 1 , statistical analyses were performed using SPSS version 21. The variable categories were analyzed using the χ² test, which was conditioned by the interpretation of the adjusted residue. The analysis of the residue is necessary to determine which category of the group presents a significant value (positive value) and to determine the level of significance for the excess occurrences through the adjusted residue; the corresponding positive value should be greater than 1.96, in which the observed value is significantly higher than the expected value. In addition, the values of χ², gl, and p were added to this interpretation. A significance level of p<0.05 was adopted for all analyses.

**Table 1:** Comparison of obstetric history and clinical outcomes related to Internet research

Obstetric history	n	Internet research	n (%)
Only vaginal deliveries	66	Yes	60 (90.9)
		No	6 (9.1)
Only cesarian deliveries	162	Yes	153 (94.4)
		No	9 (5.6)

The study was approved by the research ethics committee of *Hospital Israelita Albert Einstein* (CAAE: 69930817.3.0000.0071; # 2.160.989). The study procedures were conducted in accordance with the tenets of the Declaration of Helsinki. Informed consent was obtained from all the participants.

## RESULTS

In total, 304 questionnaires were distributed, and 302 questionnaires were returned within the stipulated time (99.3% return rate). The data relating to the participants’ baseline characteristics are presented in table 2 .

**Table 2:** Baseline characteristics of the population studied

Variables		n (%)
Age		
	<20	3 (1.0)
	20-29	16 (5.3)
	30-39	258 (85.4)
	≥40	25 (8.3)
Education		
	High school	5 (1.7)
	Bachelor's incomplete	4 (1.3)
	Bachelor's complete	237 (78.5)
	Post-graduate	5 (1.7)
	Master's degree	46 (15.2)
	Doctorate	5 (1.7)
Parity		
	1 gestation	119 (39.4)
	2 gestations	136 (45.0)
	3 gestations	30 (9.9)
	4 gestations	11 (3.6)
	5 gestations	3 (1.0)
	6 gestations	1 (0.3)
	7 gestations	1 (0.3)
	13 gestations	1 (0.3)
Obstetric history		
	Only vaginal birth	66 (0)
	Only cesarian section	162 (0)
	At least one miscarriage	59 (0)

Table 3 presents the number of internet searches. Most of the participants (92%) researched issues related to pregnancy and childbirth at least once on the Internet. Regarding frequency, 18.2% of the participants used the internet for this purpose a few times, 32.4% many times, and 24.8% did not quantify the number of internet searches they had conducted during pregnancy.

**Table 3:** Results of questionnaire

Question		n (%)
Have you done any research on the internet about pregnancy/childbirth?		
	Yes	278 (92.1)
	No	24 (7.9)
If yes, how many times?		
	Few times	55 (18.2)
	Moderate times	50 (16.5)
	Many times	98 (32.4)
	Did not quantify	75 (24.8)
Which site did you search?		
	Blogs	124 (41.0)
	Wikipedia	26 (9.0)
	Google	256 (85.0)
	Social media	104 (34.0)
	Apps	175 (58.0)
What was the reason for your research?		
	Fetal development	244 (81.0)
	Nutrition	147 (49.0)
	Childbirth	216 (72.0)
	Breastfeeding	181 (60.0)
	Obstetrics complications	126 (42.0)
	Other subjects	21 (7.0)
Have you discussed with your doctor the subjects you researched on the internet?		
	Yes	245 (88.1)
	No	33 (11.9)
Do you validate on the internet the information given by your doctor?		
	Yes	117 (42.1)
	No	161 (57.9)
What is the relevance of the information consulted on the internet in your decision making?		
	Low	44 (15.8)
	Medium	167 (60.1)
	High	59 (21.2)
	Very high	8 (2.9)

When asked about the source of the searches, 85% reported searching Google, 58% searched apps, and 41% used blogs. As for the reason for the search, fetal development was the most searched theme (81%), followed by childbirth (72%) and breastfeeding (60%). Twenty-one participants (7%) selected "other subjects," which allowed a free response; search topics given under "other subjects" included "medications," "care for the newborn," "miscarriage," "maternal and fetal diseases," "physical exercises," "puerperium," and "cosmetics" ( Table 4 ).

**Table 4:** List of other less frequent subjects searched on the Internet

Other subjects	
	Medication, baby names, exercise
	Daily routine with a newborn
	Fetal diseases
	Newborn care
	Pain and nausea
	Alternative medicine, vaccines, and puerperium
	*In vitro* fertilization
	Dealing with first born child, pregnancy weight, skin care
	Thrombophilia
	Room decoration
	Newborn clothing
	Exercises after birth, how to raise siblings
	Syndromes
	Miscarriage, maternal obstetric symptoms
	Newborn sleep
	Contractions
	Gestational diabetes

An interesting finding regarding the validation of information acquired on the Internet was that 88.1% of the participants discussed the subjects researched with their physicians and 42.1% used the internet to validate the information provided by the doctor under consultation, while 57.9% reported not validating the information provided by their physicians online.

Regarding the relevance that the participants ascribed to the information found on the Internet, 60.1% considered the information as having medium relevance, 21.2% as having high relevance, 2.9% as having very high relevance, and 15.8% as having low relevance.

Another data point was a comparison of the participants’ profiles with their delivery route and obstetric history. We compared women who only had a vaginal delivery with those who only had cesarean sections, disregarding anyone who had already had more than one route of delivery, *e.g* ., a vaginal delivery and a previous cesarean section, but there was no statistical difference by χ^2^ tests (0.329).

## DISCUSSION

This study is unprecedented in the Brazilian population for evaluating the profile of the use of obstetric information during prenatal care. Following the trend of other Brazilian studies on the subject, our study showed that the use of the internet is very prevalent in assisting pregnant women to find information about their pregnancy and puerperium.^( 1 , 19 )^

Medical content on the Internet is increasing exponentially, leading to the availability of data that were previously restricted to health professionals. This allows women to have greater knowledge and control over their health, assisting them in decision-making alongside their physicians throughout pregnancy.

One reason that has led to an increase in the search for health-related information is that health professionals need to see more patients in less time. As a result, discussions regarding diagnosis, disease, and treatments have become more superficial, forcing patients to look for other means to be informed.^( 20 )^

However, the information obtained is not always accurate. Many people search for data on websites and social media that are not linked to institutions or professionals in the area, where they often find outdated or incorrect information.^( 20 )^ In a systematic review, Daraz et al. investigated the quality of several medical content sites and concluded that there is great variation in the quality of sites when compared with the organization that published the content and the specialty involved.^( 21 )^ As for specialties, psychiatry, gynecology, and obstetrics received worse scores than anesthesiology, *e.g* . However, in general, all the scores were low.^( 21 )^

Several studies have shown in detail the discrepancies that exist between some websites and the medical literature. In a study on prostate cancer treatment, erroneous information accounted for 73% of the pages evaluated.^( 22 )^ In obstetrics, the quality of the sites was generally low, as verified in specific studies on labor analgesia.^( 23 )^

This information bias became even more evident during the health crisis generated by the COVID-19 pandemic, during which there was an explosion of false information related to coronavirus disease prevention and treatment. When information about COVID-19 was still uncertain, the role of social media became more relevant owing to the possibility of rapidly engaging the public, which proved to be superior to websites, as evidenced, *e.g* ., by the issue of hydroxychloroquine and Ivermectin which were linked to success in the treatment of the disease before being validated.^( 24 )^

To counter this problem, several instruments have been created to assess the quality of medical information in circulation. The two most widely used codes are the Health-on-the-Net Code (HONcode) and DISCERN, which were launched in 1996 to improve the quality of medical content on the internet. DISCERN consists of 15 questions that help users evaluate medical information systematically, thus creating a score.^( 25 , 26 )^ HONcode is based on eight principles that, in addition to creating a score, offer a digital certificate, if requested, by the content author.^( 27 )^ It should be noted that because of the speed at which the Internet evolves, these instruments are often rapidly outdated, and new ones need to be created in which new aspects must be incorporated.^( 20 , 28 )^

There have been many discussions about addressing this problem. A practical way to address the misuse of information is to encourage medical professionals and associations to recommend sites where people will find relevant data.^( 19 , 29 )^ In addition, there are a number of regulatory agencies that certify sites that provide consumers with good information.^( 19 , 27 )^ Modern solutions that include the use of artificial intelligence to automatically identify websites and social media with false information through various algorithms are becoming a reality and are increasingly promising control mechanisms; *e.g* ., Samuel reported excellent results with his new algorithm, MedFact, which proved superior to existing artificial intelligence tools such as HealthTrust, which was created in 2013 by Meeyoung Park for his doctoral thesis.^( 30 , 31 )^

It is worth mentioning that even when the information is correct, access to inappropriate content may cause harm to patients and caregivers, which is a potential iatrogenic effect of the Internet.^( 19 )^ Iakovou assessed the impact of information found on galactosemia and concluded that even though most of the information on the pages visited was true, parents of patients with suspected disease were mainly focused on information related to very poor prognosis, even if it was rare.^( 32 )^ This led to anxiety and depression, which could have been avoided had there been a better explanation of the data presented.^( 32 )^

Cyberchondria is a term coined in the early 2000s to describe a condition in which the excessive use of medical information may fuel anxiety and lead to hypochondria.^( 33 )^ Blackburn's study is a good example of this condition. The researchers related anxiety and uncertainty to excessive internet searches by patients with orthopedic problems. The authors concluded that it is necessary to identify and guide patients who are more likely to present with cyberchondria.^( 34 )^ In obstetrics, cyberchondria is no different; in one study, several obstetrics patients experienced anxiety when they sought information on the Internet.^( 35 )^ In addition to psychological stress, cyberchondria can lead to multiple unnecessary visits to physicians and the performance of tests and procedures that would otherwise not have been requested.^( 19 , 33 )^

In our study, approximately 90% of the participants sought information on the Internet related to their current health status. The frequency of research on obstetrics among our participants was similar to or higher than that reported in other studies.^( 1 , 19 )^ This difference can be explained by the fact that the ease of accessing the internet has increased over time, with the population becoming increasingly accustomed to and confident in searching for online information.

Compared with international studies on obstetrics, the findings presented here are similar to those observed in global literature.^( 15 , 35 )^ In Sayakhot's systematic review, several studies showed that approximately 90% of pregnant women sought information on the Internet at some point during pregnancy.^( 15 )^

Another data point from our research is the search frequency. Most patients use the internet at moderate-to-intense levels. More than 30% mentioned that they used the internet more than 100 times during pregnancy (more than 10 times a month). This finding is similar to those reported in other studies on the obstetric population.^( 10 )^ With the increase in internet access and speed, especially in portable devices, this number is expected to continue to grow. Social media and health-related apps are expected to be the main drivers of this surge. However, Google remains the main search site for information about obstetrics.^( 9 )^ In our study, Google was the primary tool used to find information, followed by apps.

The three most cited search keywords were fetal development, nutrition, and childbirth, which is similar to the findings of Sayakhot et al. and Scaioli at al.^( 15 , 36 )^

The vast majority of patients shared information found on the internet with their doctors, often to validate the truthfulness of the data. As mentioned earlier, data found on the internet are often incorrect, especially for obstetrics. According to Daraz et al., obstetrics received the lowest score in a study using HONcode.^( 21 )^ Therefore, it is encouraging to see that patients share and discuss such information with physicians, as observed in patients from other countries.^( 35 )^

Another objective of this study was to evaluate whether internet searches influenced delivery methods. Despite a slight predominance of cesarean sections, we did not observe a statistically significant difference between internet searches and the delivery route used by the participants. This could be explained by the fact that the participants ascribed low relevance to the information accessed.

## CONCLUSION

In today's connected world, seeking medical content on the Internet is a common and increasingly frequent part of people's lives. Obstetric patients are no exception; they seek medical data in different ways. Given the large amount of inaccurate information available on the internet, it is reassuring to see that a large percentage of women share the information they have accessed with their doctors, as evidenced by the fact that there is no difference in the choice of delivery route based on the characteristics of Internet searches for medical information.

## Appendix 1 Questionnaire - The use of the internet in prenatal care

**Number of pregnancies:________________ Normal delivery:________________ Cesarean delivery:________________ Abbreviations:_________________**

**Mark an (X) in the following alternatives that you deem appropriate:**
Age:

( ) < 20 years
( ) 20-29 years
( ) 30-39 years
( ) >40 years

Schooling:

( ) High school
( ) Incomplete higher education.
( ) Higher education level
( ) Master's degree
( ) Doctorate

1) Have you done any research on the internet about pregnancy/childbirth?

( ) Yes - how many times: _____
( ) No

2) Which site did you search?

( ) Blogs ( ) Google ( ) Social Media ( ) Wikipedia ( ) Applications

3) What was the reason for your research?

( ) Fetal development
( ) Nutrition during pregnancy
( ) Childbirth
( ) Breastfeeding
( ) Complications during pregnancy
( ) Other: __________

4) Have you discussed with your doctor the subjects you researched on the internet?

( ) Yes
( ) No

5) Do you validate on the internet the information given by your doctor?

( ) Yes
( ) No

6) What is the relevance of the information consulted on the internet in your decision making?

( ) Very high
( ) High
( ) Average
( ) Low
